# Deep Brain Stimulation of the Internal Globus Pallidus Improves Response Initiation and Proactive Inhibition in Patients With Parkinson’s Disease

**DOI:** 10.3389/fpsyg.2018.00351

**Published:** 2018-04-06

**Authors:** Yixin Pan, Linbin Wang, Yingying Zhang, Chencheng Zhang, Xian Qiu, Yuyan Tan, Haiyan Zhou, Bomin Sun, Dianyou Li

**Affiliations:** ^1^Department of Functional Neurosurgery, Ruijin Hospital, Shanghai Jiao Tong University School of Medicine, Shanghai, China; ^2^Department of Psychiatry, Ruijin Hospital, Shanghai Jiao Tong University School of Medicine, Shanghai, China

**Keywords:** internal globus pallidus, Parkinson’s disease, deep brain stimulation, inhibitory control, stop-signal task

## Abstract

**Background:** Impulse control disorder is not uncommon in patients with Parkinson’s disease (PD) who are treated with dopamine replacement therapy and subthalamic deep brain stimulation (DBS). Internal globus pallidus (GPi)-DBS is increasingly used, but its role in inhibitory control has rarely been explored. In this study, we evaluated the effect of GPi-DBS on inhibitory control in PD patients.

**Methods:** A stop-signal paradigm was used to test response initiation, proactive inhibition, and reactive inhibition. The subjects enrolled in the experiment were 27 patients with PD, of whom 13 had received only drug treatment and 14 had received bilateral GPi-DBS in addition to conventional medical treatment and 15 healthy individuals.

**Results:** Our results revealed that with GPi-DBS on, patients with PD showed significantly faster responses than the other groups in trials where it was certain that no stop signal would be presented. Proactive inhibition was significantly different in the surgical patients with GPi-DBS on versus when GPi-DBS was off, in surgical patients with GPi-DBS on versus drug-treated patients, and in healthy controls versus drug-treated patients. Correlation analyses revealed that when GPi-DBS was on, there was a statistically significant moderate positive relationship between proactive inhibition and dopaminergic medication.

**Conclusion:** GPi-DBS may lead to an increase in response initiation speed and improve the dysfunctional proactive inhibitory control observed in PD patients. Our results may help us to understand the role of the GPi in cortical-basal ganglia circuits.

## Introduction

Impulse control disorders have been shown to occur in approximately 17% of patients with PD who receive dopamine replacement therapy (Weintraub et al., 2010a). ICD pathology is highly heterogeneous, several factors have been independently linked to ICDs, including dopamine agonist treatment, levodopa treatment, age, being unmarried, living in the United States, a family history of gambling problems, and cigarette smoking (Weintraub et al., 2010a). Recent studies suggest that patients with PD may have a biological predisposition to ICD development that is also associated with dopaminergic pathology. However, chronic treatment with dopaminergic medications may also be an important factor in ICD development (Voon et al., 2017). Decreasing or discontinuing dopamine replacement treatment is not appropriate for many patients with PD owing to the risk of motor system deterioration or the development of dopamine agonist withdrawal syndrome (Rabinak and Nirenberg, 2010), but randomized controlled studies have shown that ICD symptoms could be decreased by adding drugs such as amantadine (Thomas et al., 2010; Weintraub et al., 2010b) and naltrexone (Papay et al., 2014), or by the use of cognitive behavioral therapy (Okai et al., 2013).

Prospective studies have shown that in patients with ICDs due to decreased or discontinued use of dopaminergic medication, DBS of the STN can improve ICD symptoms (Lhommée et al., 2012; Amami et al., 2015). This is, however, inconsistent with retrospective studies, in which the STN has been implicated in the etiology of ICDs (Lim et al., 2009; Moum et al., 2012). Subsequent investigations have suggested a potential role for the STN in inhibitory control, suggesting that deficits in STN inhibition may result in impulsive actions (Jahanshahi et al., 2015). Studies of STN-DBS in patients with PD have used several experimental tasks to determine the role of the STN in impulsivity associated with PD. For example, studies using probabilistic decision-making (Frank et al., 2007; Cavanagh et al., 2011; Coulthard et al., 2012), the SST (van den Wildenberg et al., 2006; Ray et al., 2009; Obeso et al., 2013), and the “Simon effect” task (Wylie et al., 2010) have reported an association between STN–DBS and deficits in conflict resolution, response selection under conflict, and response inhibition. Similarly, another study used diffusion-weighted MRI and functional MRI, and found a connection between the pre-SMA, IFC, and STN, in a conditional SST, suggesting the existence of a specific inhibitory network (Aron et al., 2007). Moreover, follow-up studies indicated that the STN may receive input from the IFC, resulting in a global “stop” of action through the hyperdirect pathway (Sano et al., 2013), whereas connections between the pre-SMA and striatum function to selectively or proactively stop action through the indirect pathway (Majid et al., 2013). A recent electrophysiological study (Schmidt et al., 2013) in rats trained to move rightward or leftward in a SST further identified a role of the STN in inhibition. Increased STN activity was observed during all “stop” trials, whereas increased activity in the arkypallidal (“arky”) neurons of the GPe only occurred during successful “stop” trials. This suggests that the STN provides fast “stop” signals to the substantia nigra pars reticulata (SNr), which arrive prior to signals from the striatum that indicate appropriate stop-action behavior.

Since the GPi, along with the SNr, forms the final output pathway from the BG to the cerebral cortex (Hoover and Strick, 1993), and thus is a potential target of DBS for patients with PD (Rodriguez-Oroz et al., 2005; Follett et al., 2010), its role in inhibition is of great interest. Previous evidence (Kohl et al., 2015) indicates that the GPi may be associated with response initiation. Although no changes in ICD diagnoses have been observed between pre- and post-treatment with GPi-DBS (Moum et al., 2012), the influence of GPi-DBS on inhibitory control in patients with PD is still unclear. In this study, we applied a variant of the stop-signal paradigm, a classic experimental task used to measure response inhibition (Logan, 1994), to examine the effects of GPi-DBS on response initiation, proactive inhibition, and reactive inhibition.

## Materials and Methods

### Participants

The study was approved by the Ruijin Hospital Ethics Committee of Shanghai JiaoTong University School of Medicine and carried out in accordance with the Declaration of Helsinki. All participants provided written informed consent before entering the study.

The participants were 27 patients diagnosed with idiopathic PD according to the UK PD Society Brain Bank clinical diagnostic criteria, recruited from the Ruijing Hospital Department of Functional Neurosurgery and Department of Neurology, and 15 matched healthy control subjects. Thirteen PD patients received only dopaminergic therapy and 14 PD patients received bilateral GPi-DBS in addition to dopaminergic therapy. The inclusion criteria were (a) age 55–80 years, (b) right-handed, (c) Hoehn and Yahr stage 1.5–4 with medication OFF, (d) disease duration 5–12 year, and (e) corrected-to-normal vision and hearing. The exclusion criteria were (a) secondary parkinsonism, (b) dementia, and (c) significant concurrent depression. The patients receiving GPi-DBS were examined 3–36 months post-surgery. Demographic and clinical features of all the patients and healthy controls are summarized in **Table 1**.

**Table 1:** Demographics and clinical features of PD patients and healthy control subjects (mean and standard deviations).

	GPi-DBS surgical PD patients^a^ (*n* = 14)	Drug-treated PD patients^b^ (*n* = 13)	Healthy controls^c^ (*n* = 15)	Group difference (a vs. b)	Group difference (a vs. c)	Group difference (b vs. c)
**Sex ratio (M:F)**	5:9	9:4	7:8	n.s.	n.s.	n.s.
**Age (years)**	64 (8)	66 (7)	65 (7)	n.s.	n.s.	n.s.
**Education (years)**	13 (4)	12 (5)	9 (5)	n.s.	**<0.001**	**<0.01**
**Disease duration (years)**	9 (3)	8 (3)	N/A	n.s.	N/A	N/A
**Hoehn and Yahr stage**
DBS off	2.9 (0.7)	2.2 (0.4)	N/A	**<0.001**	N/A	N/A
DBS on	2.6 (0.5)			**0.001**		
**Levodopa-equivalent daily doses (mg)**	554 (203)	733 (282)	N/A	**<0.01**	N/A	N/A

*GPi-DBS, deep brain stimulation of the internal globus pallidus; N/A, not applicable; DBS off, GPi-DBS off condition; DBS on, GPi-DBS on condition. ^*a*^GPi-DBS surgical PD patients. ^*b*^Drug-treated PD patients. ^*c*^Healthy controls. Group difference: *P*-values of chi-squared or independent sample two-tail *t*-tests, as appropriate. n.s., not significant, *P* > 0.1. *P*-values indicated significant difference were marked in bold*.

### Materials

The experimental task was controlled in MATLAB (R2014a version, The MathWorks, Inc., Natick, MA, United States) and Psychtoolbox 3. Stimuli were delivered via Dell monitor (Dell-P2317H, 23 inches, 1920 × 1080 pixels, 60 Hz refresh rate). Responses involved a key press on a Dell keyboard (Dell-KB216p). Participants were seated approximately 80 cm in front of the screen.

### Design

The study implemented a variant of the SST. *Certain-go* RTs were measured by 30 trials, and 100 *uncertain-go* trials were also performed, in which *stop*-signals followed the *go*-signals on some trials. All patients were tested in on-medication states. GPi-DBS surgical patients with PD were asked to complete this task with GPi-DBS on and off in different sessions. The two sessions were conducted 30 min apart to allow the patients to adapt to the change in stimulation condition, and the stimulation effects to dissipate. The order of GPi-DBS on or off sessions was counterbalanced across patients. Drug-treated patients with PD and healthy controls were asked to perform the task only once. Practice trials were performed until an accuracy of >90% was achieved in 10 consecutive trials, indicating that the task instructions were understood.

### Stop-Signal Task

**FIGURE 1:**
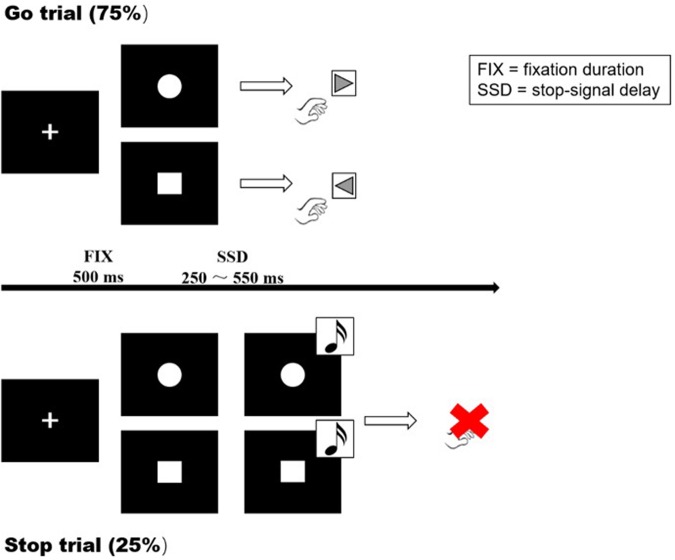
Visual representation of the *Go* task and SST. A cross was presented at the start of each trial, with a fixation duration (FIX) of 500 ms. *Go trial*: participants were instructed to respond as quickly as possible on *go* trials (left for the square, right for the circle). *Stop* trial: the participants were instructed to inhibit their responses when a *stop* signal (sound) appeared. SSD: dynamically adjusted delay between *go* and *stop* signals.

The SST is a useful tool to study response inhibition and performance monitoring. It requires participants to withhold their responses when a delayed *stop*-signal is presented. Participants were instructed to respond as quickly as possible on *go* trials and withhold responding on occasional *stop* trials. At the beginning of each trial, a fixation cross appeared in the center of the screen for 500 ms. The cross was replaced with either a circle or a square (*go* signals). On *go* trials, participants were instructed to quickly respond by pressing the left or right key (left for square, right for circle) with their index fingers. On *stop* trials, the *go* signal was followed by an unexpected tone (the *stop*-signal; 900 Hz, 85 dB, 500 ms) after a variable delay, indicating that no response should be made to the *go* signal (**Figure 1**).

Each session consisted of 30 *certain-go* trials (trials with no stop-signals) and 100 *uncertain-go* trials (75% *go* trials and 25% *stop* trials, in pseudo-random order). On *stop* trials, the delay between the *go* and *stop* signals (the SSD) ranged from 50 to 550 ms. The initial SSD was 250 ms and increased or decreased in a step-wise manner (50 ms increase following a successful stop and 50 ms decrease following each failure). Responses were required within 3250 ms, after which the trial was terminated. Stimuli were presented until a response was made, in both *go* trials or *stop* trials. At the end of each trial, a feedback statement (“correct,” “incorrect,” “success in inhibition,” “failure in inhibition,” and “response too early”) of 500 ms appeared on the screen to indicate whether the trial was successful.

### Statistical Analysis

The key parameters were accuracy on *go* trials, mean RT for correct *go* trials, RDE, and SSRT. Paired sample *t*-tests were performed to compare accuracy and mean *go* RT during GPi-DBS on and off states. Independent sample *t*-tests were used to determine differences in accuracy and mean *go* RT among all other groups.

Proactive inhibition and reactive inhibition were estimated for each stimulation condition for *stop* trials. The index of reactive inhibition as measured by the SSRT data was calculated by subtracting the mean SSD from the mean *go* RT. The index of proactive inhibition, or RDE, was obtained by subtracting the mean *certain-go* RT from the mean *uncertain-go* RTs. To determine differences in RDE and SSRTs, corresponding *t*-tests were performed.

All correlations (levodopa-equivalent daily doses versus RDE within groups) were estimated by Pearson’s linear correlation coefficient.

Statistical analyses of the data were performed with SPSS 20 (IBM, White Plains, NY, United States). Tests with *P* < 0.05 (one-tailed according to the proposed unidirectional hypotheses) were treated as significant.

## Results

### Go Reaction Times

**FIGURE 2:**
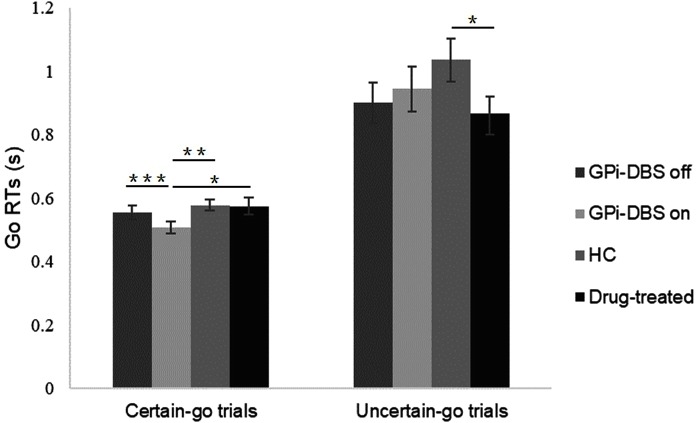
Mean *Go* RTs for patients with PD with pallidal DBS on or off, patients who only underwent drug treatment (drug-treated), and healthy controls (HCs) in the *certain-go* trials and *uncertain-go* trials. ^∗^Indicates significant differences with ^∗∗^ representing *P*-value < 0.01 and ^∗∗∗^ representing *P*-value < 0.001. Error bars indicate standard error of the mean (SEM). All *t*-tests tested significance with a one-tailed test.

As shown in **Figure 2**, in GPi-DBS surgical patients with PD the *certain-go* RTs were significantly shorter in the GPi-DBS-on condition than in the GPi-DBS-off state [*t*(13) = 5.465, *P* < 0.001]. *Certain-go* RTs were also significantly shorter in the surgical PD patients with GPi-DBS-on than in the healthy controls [*t*(27) = -2.693, *P* = 0.006] and drug-treated patients [*t*(25) = -2.071, *P* = 0.024]. This indicates an immediate improvement in the performance of *certain-go* trials when GPi-DBS was turned on.

For *uncertain-go* trials, only one significant difference in RTs was observed: the *uncertain-go* RTs were significantly shorter in the drug-treated PD patients than in the healthy controls [*t*(26) = 1.947, *P* = 0.031]. No other significant differences were observed in the *certain-go* trials or in the *uncertain-go* trials (*P* > 0.05).

### Accuracy

**FIGURE 3:**
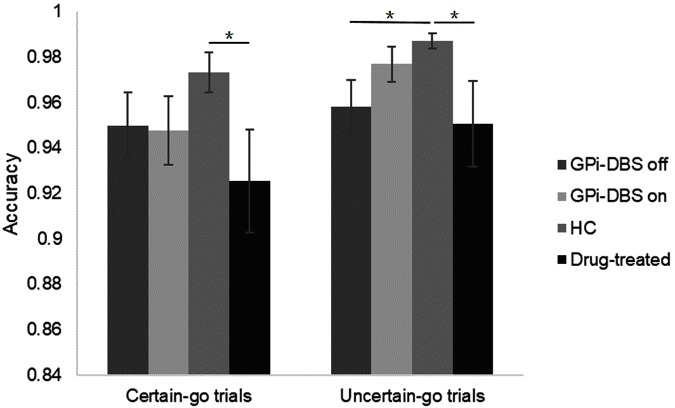
Accuracy for patients with PD with GPi-DBS on or off, patients who only underwent drug treatment (drug-treated), and HCs in the *certain-go* trials and *uncertain-go* trials. ^∗^Indicates significant differences. Error bars indicate SEM. All *t*-tests tested significance with a one-tailed test.

We observed a significant difference in accuracy between the drug-treated PD patients and healthy controls in the *certain-go* trials [*t*(26) = 2.068, *P* = 0.024]. Specifically, the healthy controls reacted slightly more correctly than the drug-treated patients. On the *uncertain-go* trials, accuracy was significantly greater in the healthy controls than in the drug-treated patients [*t*(26) = 2.029, *P* = 0.026] and also greater than in the GPi-DBS surgical patients with PD in the GPi-DBS off condition [*t*(27) = 2.426, *P* = 0.011]. No differences in accuracy were seen for any other comparisons between groups (*P* > 0.05) (**Figure 3**).

### Reactive Inhibition (SSRT)

**FIGURE 4:**
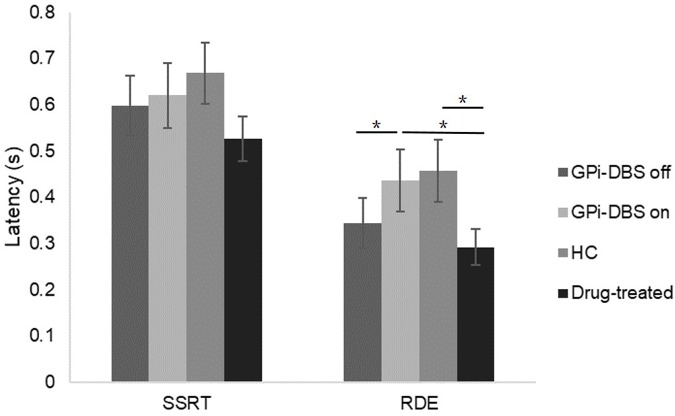
SSRTs and RDE for patients with PD with GPi-DBS on or off, patients who only underwent drug treatment (drug-treated), and HCs in the *uncertain-go* trials. ^∗^Indicates significant differences. Error bars indicate SEM. All *t*-tests tested significance with a one-tailed test.

Response inhibition was measured by mean SSRT (**Figure 4**). No significant differences in SSRTs were observed among the groups (*P* > 0.05).

### Proactive Inhibition

A significant difference in proactive inhibition was observed within the surgical PD patients when comparing the GPi-DBS on condition with the off condition [*t*(13) = 2.570, *P* = 0.012]. Proactive inhibition was also significantly different between the surgical patients with GPi-DBS on and the drug-treated patients [*t*(25) = 1.820, *P* = 0.040]. These results suggest that surgical patients in the GPi-DBS off state and drug-treated patients engage in significantly less proactive inhibition than surgical patients when their GPi-DBS is on.

There was a significant difference in proactive inhibition between the healthy controls and drug-treated PD patients as well [*t*(26) = 2.025, *P* = 0.027]. Furthermore, despite the fact that the difference between the healthy controls and surgical patients with GPi-DBS off was not significant (*P* > 0.05), a lower RDE value was obtained in the surgical patients with GPi-DBS off than in the healthy controls. This may be taken to suggest that the healthy controls showed more proactive inhibition than the drug-treated patients and surgical patients with GPi-DBS off.

No significant differences in proactive inhibition were observed between the surgical PD patients with GPi-DBS on and the healthy controls, or for any other comparisons (*P* > 0.05).

### Correlations Between Levodopa-Equivalent Daily Doses and Proactive Inhibition

**FIGURE 5:**
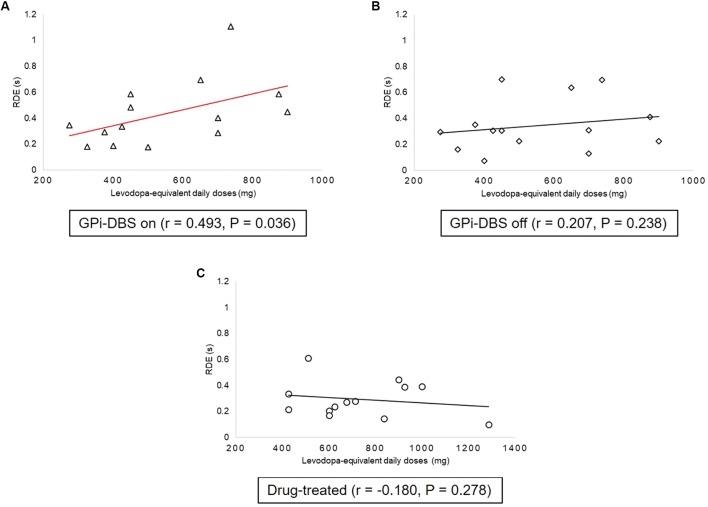
Correlations between proactive inhibition and levodopa equivalent daily dose within groups: patients with PD with GPi-DBS on or off, patients who only underwent drug treatment (drug-treated). **(A)** Correlations between proactive inhibition and levodopa-equivalent daily doses in patients with PD with GPi-DBS on. **(B)** Correlations between proactive inhibition and levodopa-equivalent daily doses in patients with PD with GPi-DBS off. **(C)** Correlations between proactive inhibition and levodopa-equivalent daily doses in drug-treated patients. All Pearson’s correlations tested significance with a one-tailed test. Line marked with red indicates significant correlation.

Correlation analyses were carried out between levodopa-equivalent daily doses and proactive inhibition within groups (**Figure 5**). There was a statistically significant, moderate positive relationship between proactive inhibition and levodopa-equivalent daily doses in the surgical PD patients with GPi-DBS on [*r* = 0.493, *P* = 0.036]. This indicates that when GPi-DBS was turned on, the more dopaminergic medication the surgical patients took, the more proactive inhibition they displayed. However, in the surgical PD patients with GPi-DBS off and in the drug-treated patients, the correlations were not significant (*P* > 0.05).

## Discussion

In this study, we investigated two types of response inhibition in patients with PD after GPi-DBS: proactive and reactive inhibition. Many earlier studies explored the role of the STN in proactive and reactive inhibition, and many were conducted with surgical PD patients with STN-DBS using the stop-signal paradigm (van den Wildenberg et al., 2006; Ray et al., 2009; Obeso et al., 2013). In contrast, studies of the impact of GPi-DBS on inhibition have been few. Recently, a study found that GPi-DBS could significantly alter the speed of response initiation but not reactive inhibition (Kohl et al., 2015). However, due to the limited sample size and lack of a control group of drug-treated patients, the conclusions needed to be further verified. The results of our experiments (**Table 2**) indicate that with GPi-DBS activated, patients with PD showed significantly faster *certain-go* RTs than the other groups, which suggests that the speed of response initiation can be increased by GPi-DBS. However, no differences were seen in SSRTs in drug-treated or GPi-DBS surgical PD patients, indicating that these treatments may not have had any effect on reactive inhibition. Proactive inhibition, the preparatory slowing of response in the anticipation of an upcoming stop-signal (Aron, 2011), differed significantly in surgical patients with GPi-DBS on versus off, in surgical patients with GPi-DBS on versus drug-treated patients, and in healthy controls versus drug-treated patients. Combining these findings with the finding that there was a recognizably but non-significantly lower RDE for the healthy controls than for the surgical patients with GPi-DBS off, it can be speculated that GPi-DBS may reverse the abnormal proactive inhibition in the drug-treated patients and the surgical patients with GPi-DBS off. The results were further expanded by the findings from correlation analyses. The analyses showed that when GPi-DBS was on, there was a statistically significant, moderately positive relationship between proactive inhibition and dopaminergic medication in the surgical patients, indicating mixed effects of GPi-DBS and medications on proactive inhibition.

**Table 2:** Measures obtained from the SST for PD patients and healthy control subjects (mean and standard deviations).

	PD patients with GPi-DBS on^a^	PD patients with GPi-DBS off^b^	Drug-treated PD patients^c^	Healthy controls^d^	*P*-value (a vs. b)	*P*-value (a vs. c)	*P*-value (a vs. d)	*P*-value (b vs. c)	*P*-value (b vs. d)	*P*-value (c vs. d)
**Go RTs (s)**


*Certain-go* trails	0.51 (0.08)	0.55 (0.08)	0.57 (0.10)	0.58 (0.07)	**0.000^∗∗∗^**	**0.024^∗^**	**0.006^∗∗^**	0.183	0.084	**0.024^∗^**


*Uncertain-go* trials	0.94 (0.27)	0.90 (0.24)	0.87 (0.18)	1.04 (0.26)	0.156	0.200	0.179	0.349	0.077	**0.031^∗^**


**Accuracy**


*Certain-go* trails	0.95 (0.06)	0.95 (0.05)	0.93 (0.08)	0.97 (0.03)	0.425	0.211	0.073	0.183	0.084	**0.024^∗^**


*Uncertain-go* trials	0.98 (0.03)	0.96 (0.04)	0.95 (0.07)	0.99 (0.01)	0.078	0.100	0.112	0.371	**0.011^∗^**	**0.026^∗^**


**Latency (s)**


SSRT	0.62 (0.26)	0.60 (0.24)	0.53 (0.17)	0.67 (0.26)	0.310	0.145	0.310	0.195	0.223	0.051


RDE	0.44 (0.25)	0.34 (0.20)	0.29 (0.14)	0.46 (0.26)	**0.012^∗^**	**0.040^∗^**	0.415	0.224	0.104	**0.027^∗^**

*GPi-DBS, deep brain stimulation of the internal globus pallidus; on, on condition; off, off condition. ^*a*^PD patients with GPi-DBS on. ^*b*^PD patients with GPi-DBS off. ^*c*^Drug-treated PD patients. ^*d*^Healthy controls. *P*-value, *P*-values of independent sample one-tail *t*-tests or paired sample one-tail *t*-tests, as appropriate. ^∗^Indicates significant differences and they were all marked in bold*.

### Response Initiation

Our results showed a significant speedup in response initiation when GPi-DBS was on, indicating an acute facilitation by GPi-DBS of response initiation. This is consistent with the results of previous studies (Schubert et al., 2002; Kohl et al., 2015). Evidence from animal studies has shown that both the direct and indirect pathways are active during response initiation (Cui et al., 2013). While the direct pathway implements the *go* process, the indirect pathway implements the *no-go* process. The SNr/GPi receive projections from both the direct and indirect pathways and integrate the outcome of the competition between them (Afsharpour, 1985; Parent and Hazrati, 1995). Unlike the selective effects of the SNr on successful and failed stopping, the GPi neuronal activity is unselective (Schmidt et al., 2013). The lack of a specific mechanism to cancel an action, such as the phasic late excitation in the SNr (Sano et al., 2013), makes the GPi neuronal activity more suitable for generating *go* signals. This conjecture is supported by evidence from imaging studies, which show a critical activation of GPi during *go* trials (Aron, 2006). Furthermore, it has been suggested that GPi-DBS might speed response initiation by reducing the response threshold (Kohl et al., 2015). Some studies have attributed the accelerated response initiation to the net inhibitory effect of DBS on GPi activity (Dostrovsky et al., 2000; Lafreniere-Roula et al., 2010), which reduces inhibitory output to thalamo-cortical areas, and increases thalamic and cortical activation (Limousin et al., 1997), while lowering BG modulation and response thresholds.

### Reactive Inhibition

Consistent with previous studies, GPi-DBS did not affect reactive inhibition. In contrast, most studies suggested that STN-DBS impairs the normal STN activity that generates fast reactive inhibition (Ray et al., 2009; Obeso et al., 2013, 2014). Unlike the STN, the GPi is not involved in the race between the go and stop processes, and has no selective influence on go and stop signals received from the hyperdirect, direct, and indirect pathways (Schmidt et al., 2013). Moreover, our results did not indicate differences in reactive inhibition between controls and drug-treated PD patients. However, a relationship was suggested between inhibitory control and medication treatment in the previous studies. A cross-sectional study of 3090 patients confirmed the role of dopaminergic treatment in ICDs (Weintraub et al., 2010a), and another study with an integrated stop-signal and no-go paradigm confirmed the role of serotonergic treatments in reactive inhibition (Ye et al., 2014). Lower medication doses were the main limitation of our study, as a survey in Shanghai showed a lower incidence of ICDs in PD compared to that in western countries (Wang et al., 2016). The small number of trials might also compromise our interpretations. Specifically, our results showed that RTs were almost twice as long in the *uncertain-go* trials than *certain-go* trials, while the value of mean RTs was about the same on *uncertain-go* trials and *certain-go* trials in the previous studies (Verbruggen et al., 2004). This indicates the participants of the present study acted extremely cautiously in the SST, which may be the major reason why no effects on reactive inhibition were obtained. So future studies with more trials are necessary to ascertain the effect of GPi-DBS on reactive inhibition.

### Proactive Inhibition

In our experiments, while the participants responded quickly on *certain-go* trials, there was a sharp decline in response initiation speed on *uncertain-go* trials, which implies preparation for stopping, or proactive inhibition. In the present study, both global proactive inhibition and selective proactive inhibition were probably involved. The broad effect of the STN on the SNr/GPi was considered to be associated with global proactive inhibition, which might be implemented by the hyperdirect pathway (Nambu et al., 2002). A study applying transcranial magnetic stimulation and fMRI found that the pre-SMA, striatum, and pallidum were activated during selective proactive inhibition, which might be implemented by the indirect pathway (Majid et al., 2013). Dorsolateral prefrontal cortex activation has also been observed in some studies, reflecting the role of working memory in proactive inhibition (Hester et al., 2004; Vink et al., 2014).

Our results revealed abnormal proactive inhibition in the drug-treated patients and surgical patients with GPi-DBS off compared to the healthy controls. This is consistent with previous studies, which indicated proactive inhibition might be endogenously impaired in PD patients as a result of specific hypoactivation of a proactive inhibitory network (Criaud et al., 2016). However, a mixed effect of medications and PD pathology would be a more reasonable interpretation of the results, as all the participants were using medication and robust evidence has confirmed the role of Parkinson’s medications in inhibitory control (Voon et al., 2017).

Given that the GPi was crucial for both global proactive inhibition and selective proactive inhibition, and such a role was revealed by our experiments on GPi-DBS surgical patients. Since there was no difference between the surgical patients with GPi-DBS on and the healthy controls, it can be assumed that the reduced proactive inhibition implicit in the drug-treated patients and surgical patients with GPi-DBS off reverted to the normal level with GPi-DBS stimulation. Furthermore, according to the correlation analyses for medication and proactive inhibition, GPi-DBS stimulation might have interacted with medication, which could also be influenced by GPi-DBS, conjointly affecting proactive inhibition. The important role of GPi in the proactive inhibitory networks and neuronal activity changes of GPi caused by DBS stimulation most likely led to the restorative effects of GPi-DBS on proactive inhibition. Notably, the STN is also critically located in the proactive inhibitory networks, and many studies suggested that STN-DBS might affect proactive inhibition (Favre et al., 2013; Majid et al., 2013; Benis et al., 2014). Indeed, some authors concluded that DBS of STN improved proactive inhibitory control (Favre et al., 2013; Majid et al., 2013), while one study related higher levels of beta-activity in the STN to proactive inhibition (Benis et al., 2014).

### Similar Mechanisms Underlie the Changes in Response Initiation and Proactive Inhibition

High-frequency electrical stimulation of the GPi-DBS was initially thought to have an inhibitory effect on the target nucleus (Chiken and Nambu, 2015). Evidence from PD patients (Dostrovsky et al., 2000) and animal models of non-human primates (Boraud et al., 1996; Wu et al., 2001; Lafreniere-Roula et al., 2010) showed that GPi-DBS could reduce the firing rates of neighboring neurons. However, recent studies have shown that GPi-DBS not only activates inhibitory inputs from the striatum and GPe, but also activates excitatory inputs from the STN (Chiken and Nambu, 2013). This means that when an inhibitory signal from the direct pathway reaches the GPi, GPi-DBS acts as an amplifier, reducing inhibitory output to thalamo-cortical areas. This is consistent with the previous assumption with regard to the accelerating effect of GPi-DBS on response initiation. Similarly, when excitatory signals arrive from the hyperdirect and indirect pathways, GPi-DBS increases the inhibitory output and improves the dysfunctional proactive inhibitory control observed in patients with PD. Thus, the effects of GPi-DBS on response initiation and proactive inhibition may both result from the activation of afferent axons in the GPi.

### Limitations

Our study has some limitations that need to be clarified. We attempted to recruit participants with adequate cognitive capacity to complete all experiments, possibly resulting in selection bias. Furthermore, this study included fewer trials than previous studies (Verbruggen et al., 2004; Hommel et al., 2011) due to considerations related to time efficiency, which may have led to increased intra-individual variability. More trials are needed in future studies to measure the effects of GPi-DBS on reactive inhibition. Also, a longitudinal design may provide additional insight to the findings of this cross-sectional study. Lastly, levodopa-equivalent daily doses between the post-DBS group and non-surgical group were not matched; dosage of medication may therefore be a confounding factor in this study.

## Conclusion

In summary, GPi-DBS may lead to an increase in response initiation speed and improve the dysfunctional proactive inhibitory control observed in PD patients. Our results may help us to understand the role of the GPi in cortical-BG circuits. Although GPi-DBS is clinically considered a target that subtly influences cognition, its effects on some important cognitive functions such as inhibitory control warrant further investigation.

## Availability of Data and Materials

The materials described in the manuscript, including all relevant raw data, will be freely available to any scientist wishing to use them for non-commercial purposes, without breaching participant confidentiality and without conflicting with our further research.

## Ethics Statement

The study was approved by the Ethics Committee of Ruijin Hospital. Written and informed consent were obtained from all participants and the guidelines of the Helsinki Declaration were scrupulously followed.

## Author Contributions

YP, LW, CZ, and DL designed the study. LW and CZ drafted the manuscript. LW, YP, YZ, YT, XQ, and HZ acquired the data. LW, YP, and DL contributed to the data interpretation. All the authors read and approved the final manuscript.

## Conflict of Interest Statement

The authors declare that the research was conducted in the absence of any commercial or financial relationships that could be construed as a potential conflict of interest.

## Abbreviations

BG: basal ganglia
DBS: deep brain stimulation
GPe: external globus pallidus
GPi: internal globus pallidus
ICDs: impulse control disorders
IFC: right inferior frontal cortex
MRI: magnetic resonance imaging
PD: Parkinson’s disease
RDE: response delay effect
RTs: reaction times
SMA: supplementary motor area
SNr: substantia nigra reticulate
SSD: stop-signal delay
SSRTs: stop-signal reaction times
SST: stop-signal task
STN: subthalamic nucleus.
